# Editorial for the Special Issue on Graphene-Nanocomposite-Based Flexible Supercapacitors

**DOI:** 10.3390/mi15080979

**Published:** 2024-07-30

**Authors:** Prashant Shivaji Shewale, Kwang-Seok Yun

**Affiliations:** Department of Electronic Engineering, Sogang University, Seoul 04107, Republic of Korea

The evolution of hybrid materials has revolutionized the field of material science, particularly with the development of nanocomposites. Among the developed nanocomposites, graphene and its derivatives have emerged as key components due to their exceptional electronic, mechanical, and chemical properties. Graphene, a single layer of carbon atoms arranged in a two-dimensional honeycomb lattice, exhibits remarkable conductivity and a high surface area, high mechanical strength, and high flexibility [1]. These attributes make graphene an ideal candidate for enhancing the performance of nanocomposites.

The Special Issue of *Micromachines*, entitled “Graphene-Nanocomposite-Based Flexible Supercapacitors”, brings together innovative research that addresses the challenges and potentials of these advanced materials in energy storage applications. Hybrid materials combine the properties of two or more components to create a synergistic effect that surpasses the capabilities of the individual constituents. Graphene nanocomposites, in particular, have shown great promise in electrochemical energy storage [2,3] and gas detection [4] due to graphene’s ability to provide a conductive matrix that supports the uniform distribution and stability of other active materials.

The challenge lies in synthesizing these nanocomposites to maintain high conductivity, electrochemical activity, and surface area while ensuring mechanical flexibility, which is critical for wearable devices such as supercapacitors [3]. Researchers are exploring various strategies to integrate graphene with other materials, such as metal oxides, conducting polymers, and other carbon-based materials, to optimize the performance of flexible supercapacitors [5,6,7,8].

The Special Issue showcases several fascinating studies that delve into the different synthesis methods, structural characterizations, and performance evaluations of graphene-based nanocomposites. These studies highlight innovative approaches to enhance the capacitance, charge–discharge efficiency, and mechanical resilience of supercapacitors, paving the way for next-generation energy storage devices that are both high-performing and adaptable to flexible and wearable technologies.

One notable study, conducted by Nujud Mohammed Badawi et al., presents the development of highly conductive and reusable cellulose hydrogels for supercapacitor applications [9]. The research focuses on Na-Alginate-based hydrogels enhanced with poly(3,4-ethylene dioxythiophene) (PEDOT): poly(4-styrene sulfonic acid) (PSS) and dimethyl sulfoxide (DMSO). The resulting hydrogel exhibits a remarkable specific capacitance of 312 F/g, high ionic conductivity, and excellent electrochemical stability, retaining 92.5% of its capacity after 3000 cycles. The hydrogels’ conductivity was improved through in situ synthesis and H_2_SO_4_ treatment, with structural and surface analyses conducted using Fourier-transform infrared spectroscopy (FTIR) and field emission scanning electron microscopy (FESEM). Additionally, the hydrogels demonstrated excellent flexibility and self-healing properties, crucial for advanced energy storage systems. This innovative approach highlights the potential of flexible and self-healing hydrogels in enhancing the performance and longevity of supercapacitors.

A notable contribution to the present Special Issue is a study detailing the development of a highly uniform, multi-layered reduced graphene oxide/poly-2-aminobenzene-1-thiol (R-GO/P2ABT-ML) nanocomposite for symmetric supercapacitors [10]. Mohamed Rabia et al. successfully synthesized and characterized the above nanocomposite, enhancing its performance through the incorporation of a porous ball structure of polypyrrole (PB-Ppy). This addition significantly boosted the nanocomposite’s specific capacitance from 19.6 F/g to 92 F/g and its energy density from 1.18 Wh/kg to 5.43 Wh/kg. The incorporation of PB-Ppy also improves the stability of the supercapacitor, demonstrating impressive retention coefficients of 95.6% after 100 cycles and 85.0% after 500 cycles. These advancements highlight the R-GO/P2ABT-ML/PB-Ppy nanocomposite’s potential for commercial energy storage applications, thanks to its enhanced performance, cost-effectiveness, and ease of fabrication. This work underscores the promising future of such nanocomposites in advancing energy storage.

In a noteworthy study, Nujud Badawi Mohammed et al. present the development of natural solid-state hydrogel electrolytes composed of 3D pure cotton/graphene composites for supercapacitor applications [11]. Their research highlights how the incorporation of graphene into the cotton-based hydrogel significantly boosts its ionic conductivity to 13.9 × 10^−3^ S/cm at 25 °C. This advanced hydrogel demonstrates remarkable electrochemical performance, with a specific capacitance reaching 327 F/g at 3 mV/s and 385.4 F/g at 100 mA/g. The enhancement in performance is due to graphene’s role in providing smooth pathways for efficient charge carrier transport. The hydrogel also shows excellent flexibility and stability, crucial for the development of next-generation supercapacitors. The results of X-ray diffraction and morphology analyses confirm the effective dispersion and integration of graphene within the hydrogel matrix. Notably, the hydrogel successfully powered a red LED, underscoring its potential for use in portable and wearable electronics. This research emphasizes the promising capabilities of cotton/graphene-based hydrogels in advancing supercapacitor technology.

In a leading study, Mohamed Rabia et al. developed a flower-shaped cobalt sulfide–cobalt oxide/graphitic carbon nitride (CoS-Co_2_O_3_/G-C3N4) nanocomposite for symmetric supercapacitors, examining its performance in both basic and acidic mediums [12]. The cobalt sulfide–cobalt oxide/graphitic carbon nitride (CoS-Co_2_O_3_/G-C3N4) composite was synthesized using a hydrothermal method and characterized through X-ray Diffraction (XRD), X-ray Photoelectron Spectroscopy (XPS), Scanning Electron Microscopy (SEM), and Transmission Electron Microscopy (TEM), which confirmed the composite’s chemical structure and morphology. The composite features flower-like nano appendages on G-C3N4 sheets, with G-C3N4 exhibiting 2D nanosheets of approximately 80 nm in width and 170 nm in length. Electrochemical testing revealed that the supercapacitor demonstrates a specific capacitance of 361 F/g in a basic medium and 92 F/g in an acidic medium, with energy densities of 28.7 and 30.2 Wh/kg, respectively. The device shows impressive stability, retaining 100% of its performance after 600 cycles and 98.5% after 1000 cycles. This work underscores the potential of the CoS-Co_2_O_3_/G-C3N4 nanocomposite for high-capacity, stable, and cost-effective supercapacitor applications.

In a significant advancement, Amira Ben Gouider Trabelsi et al. developed a petal-like NiS-NiO/G-C3N4 nanocomposite for high-performance symmetric supercapacitors [13]. The G-C3N4 was synthesized through the combustion of urea, while the NiS-NiO/G-C3N4 composite was prepared via hydrothermal methods. Characterization techniques including SEM, XRD, and XPS confirmed the successful formation and morphology of the nanocomposite, which features petal-like NiS-NiO nanoparticles on G-C3N4 sheets. Electrochemical testing, conducted in a 0.6 M HCl electrolyte, revealed that the supercapacitor exhibits exceptional performance with a specific capacitance of 766 F/g and an energy density of 23.55 Wh/kg. Additionally, the charge–discharge time reached 790 s at 0.3 A/g, highlighting the material’s efficient energy storage capabilities. This work underscores the potential of the NiS-NiO/G-C3N4 nanocomposite for advanced supercapacitor applications, paving the way for future commercial development.

The articles included in this Special Issue highlight the transformative potential of graphene nanocomposite materials in the field of flexible supercapacitors. The innovative approaches and significant findings presented underscore the ongoing efforts to enhance the performance, stability, and practical application of these materials in energy storage technologies. The synthesis techniques and engineering strategies discussed are paving the way for the development of flexible/stretchable supercapacitors that can be integrated into wearable technology. Moreover, the exploration of novel materials and hybrid composites continues to address the challenges of conductivity, electrochemical activity, and mechanical flexibility, essential for the advancement of next-generation energy storage devices.

In summary, the Special Issue “Graphene-Nanocomposite-Based Flexible Supercapacitors” of the journal *Micromachines* provides a comprehensive overview of the current developments and future directions in this dynamic field. The contributions not only expand the scientific understanding of graphene nanocomposites but also lay the groundwork for their practical applications in wearable and flexible electronic devices.
